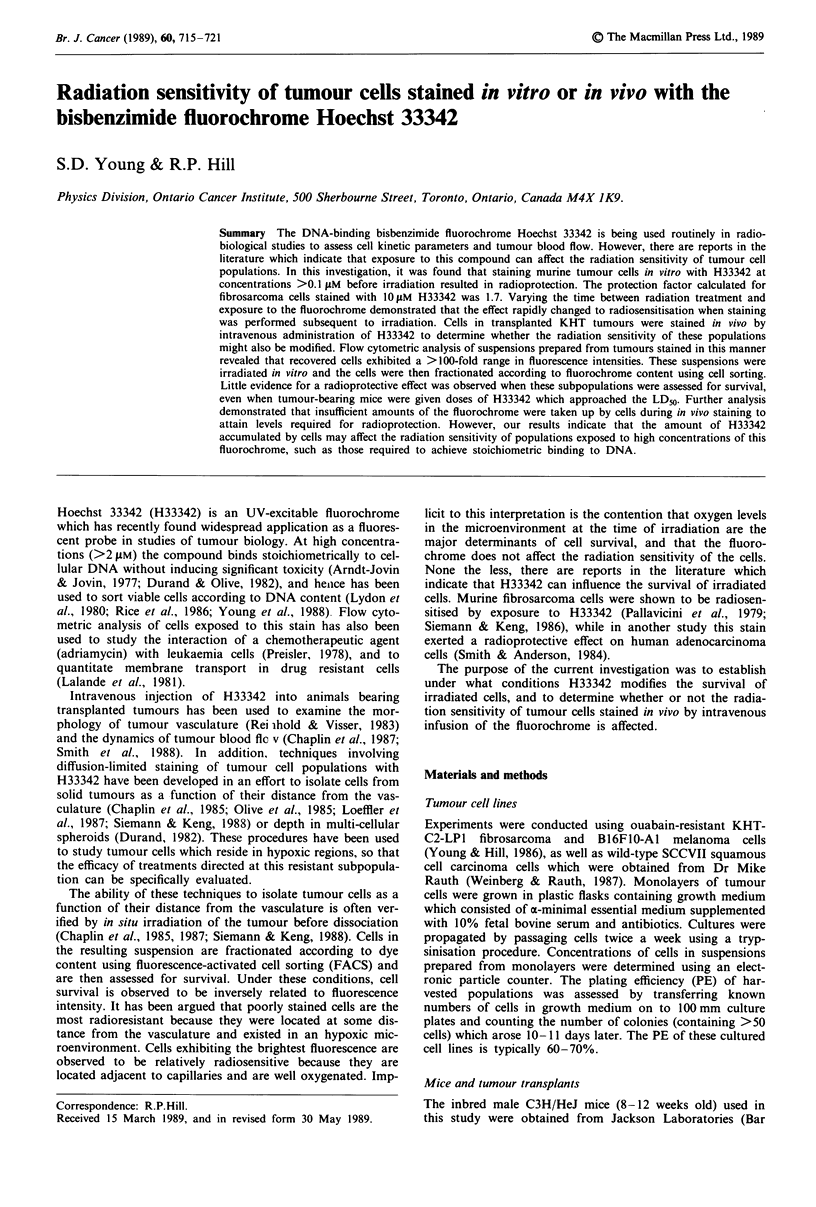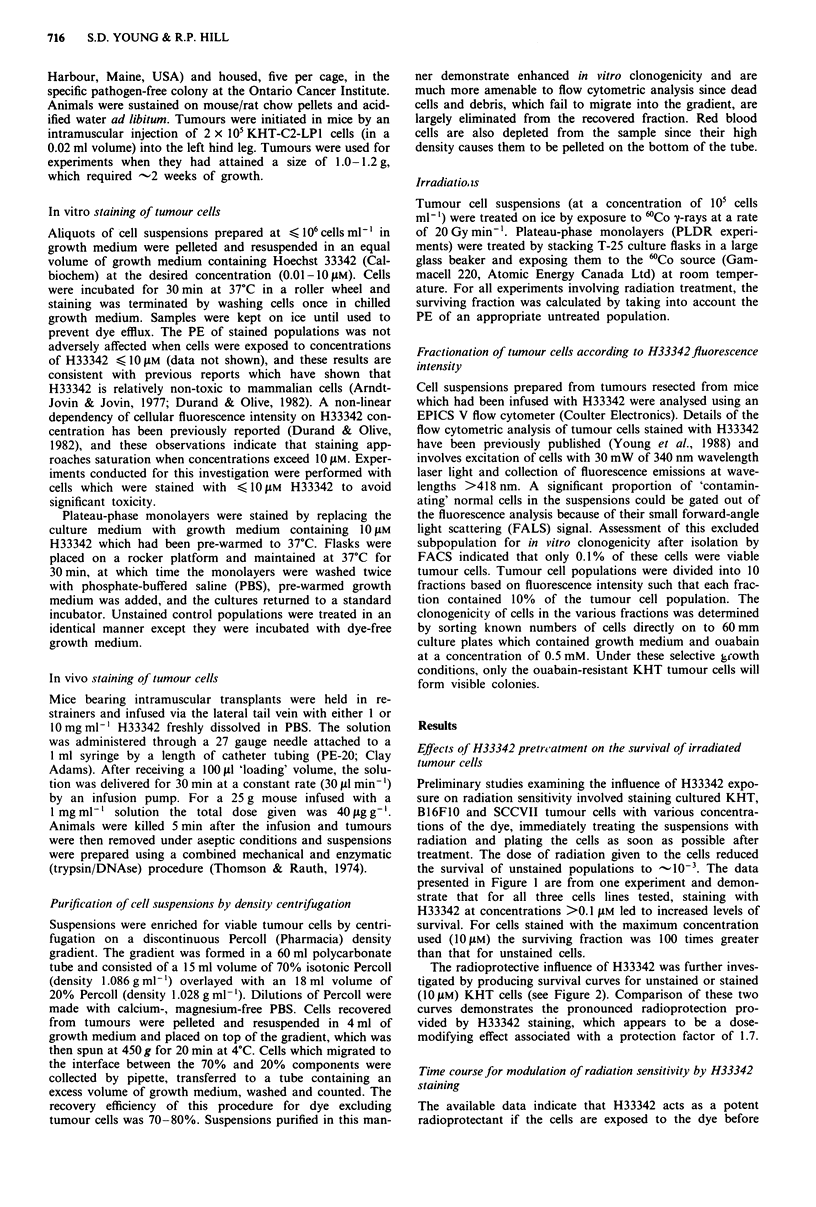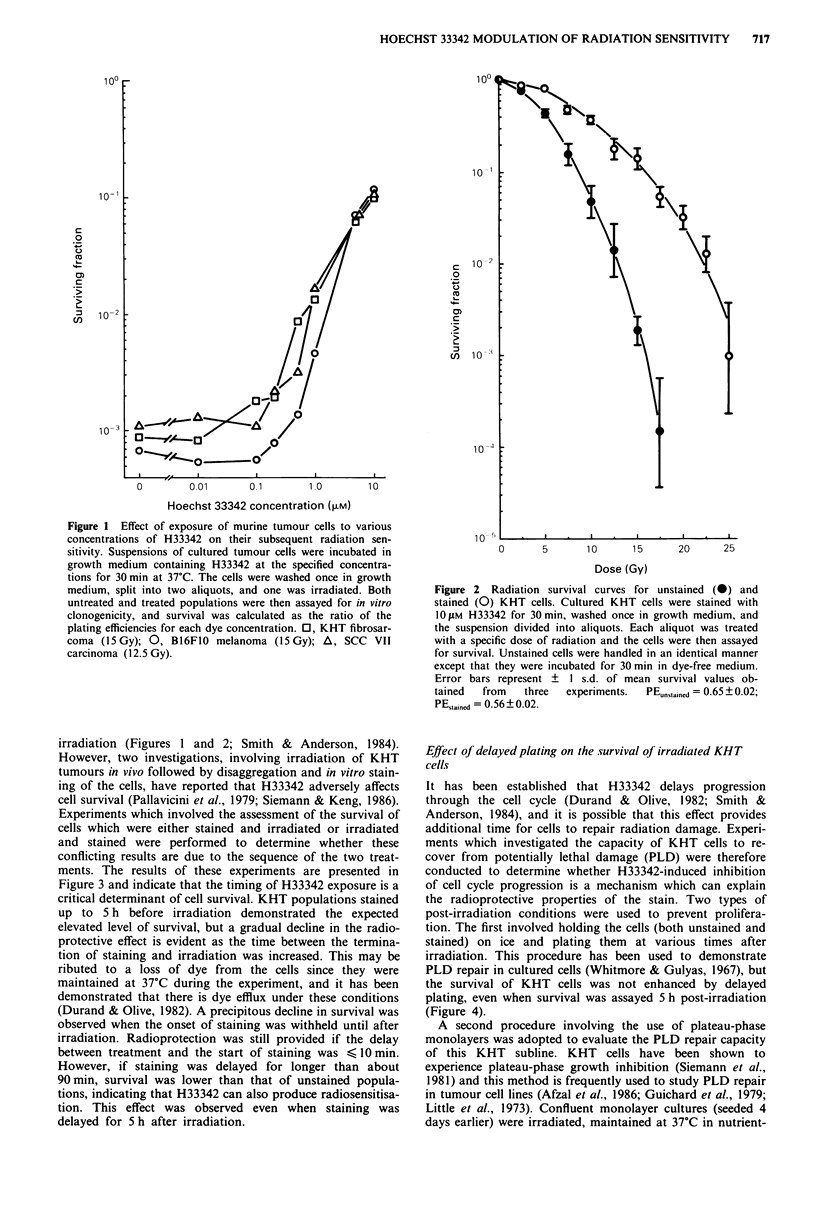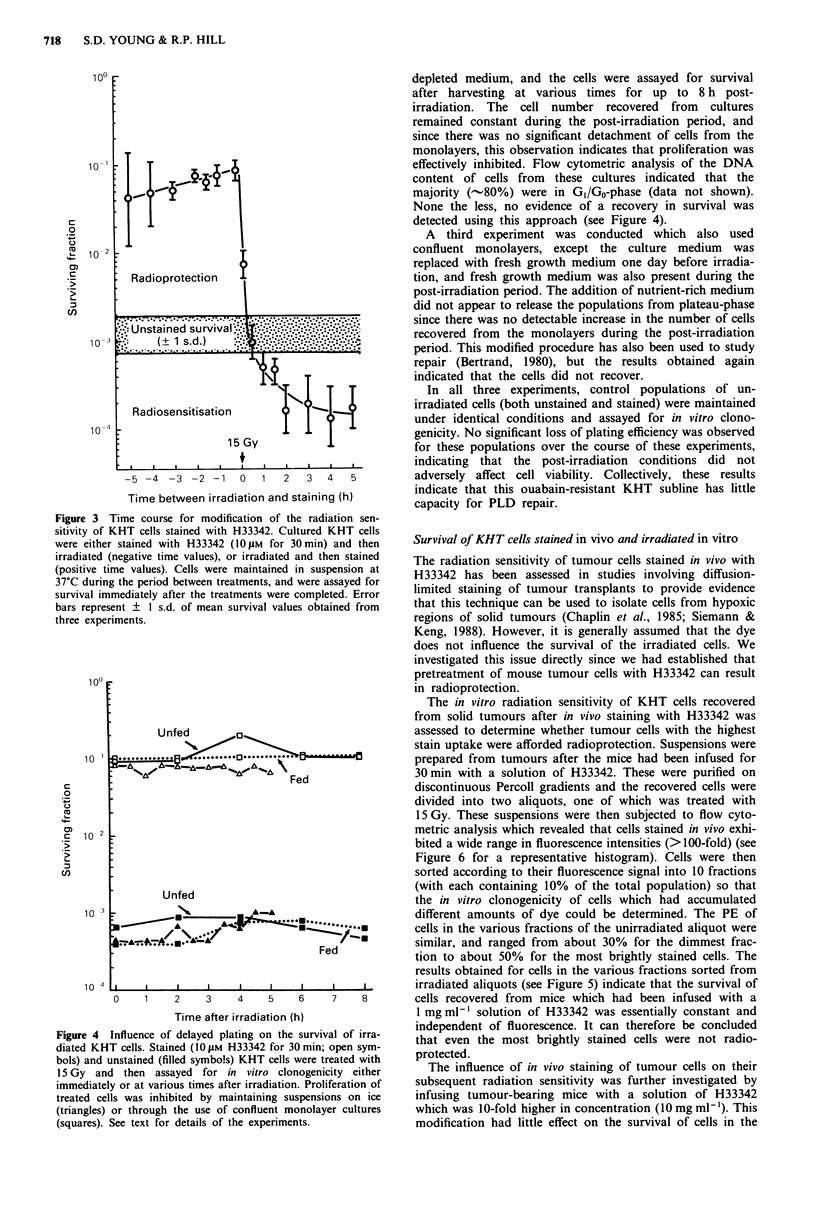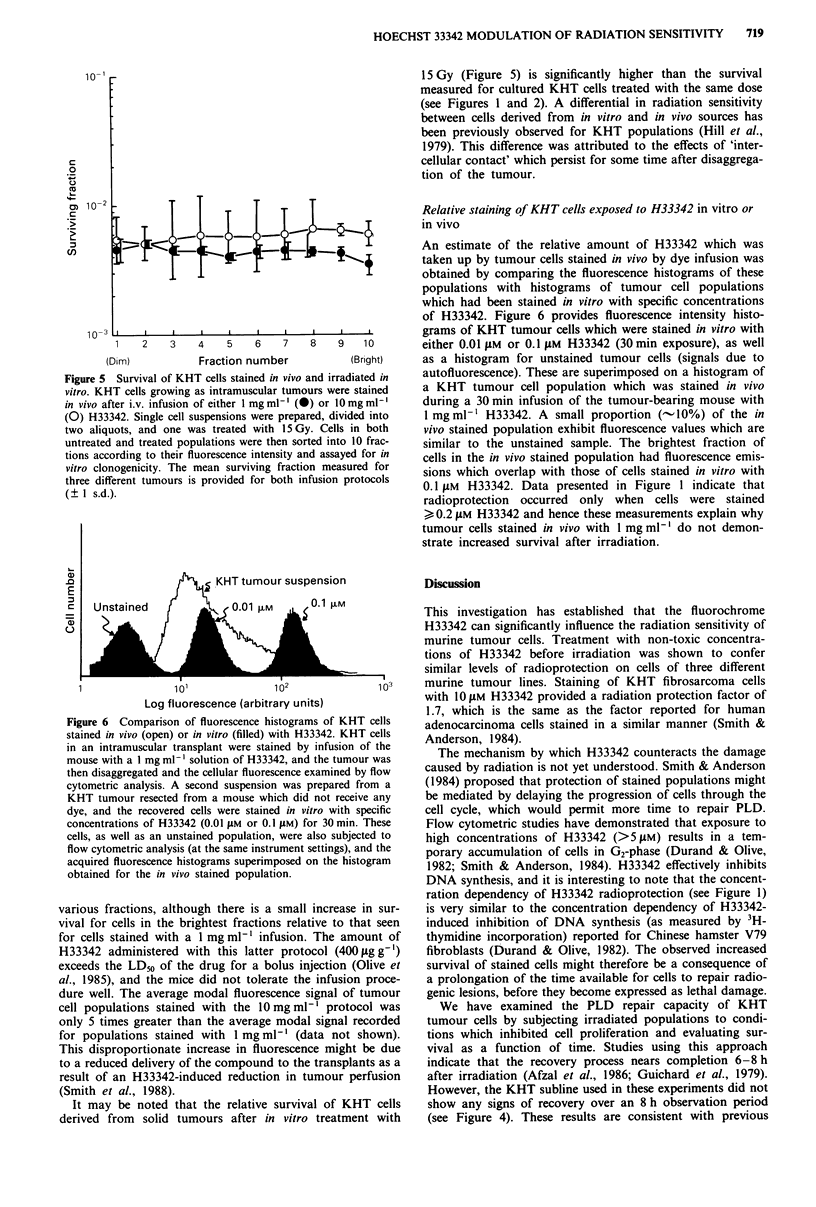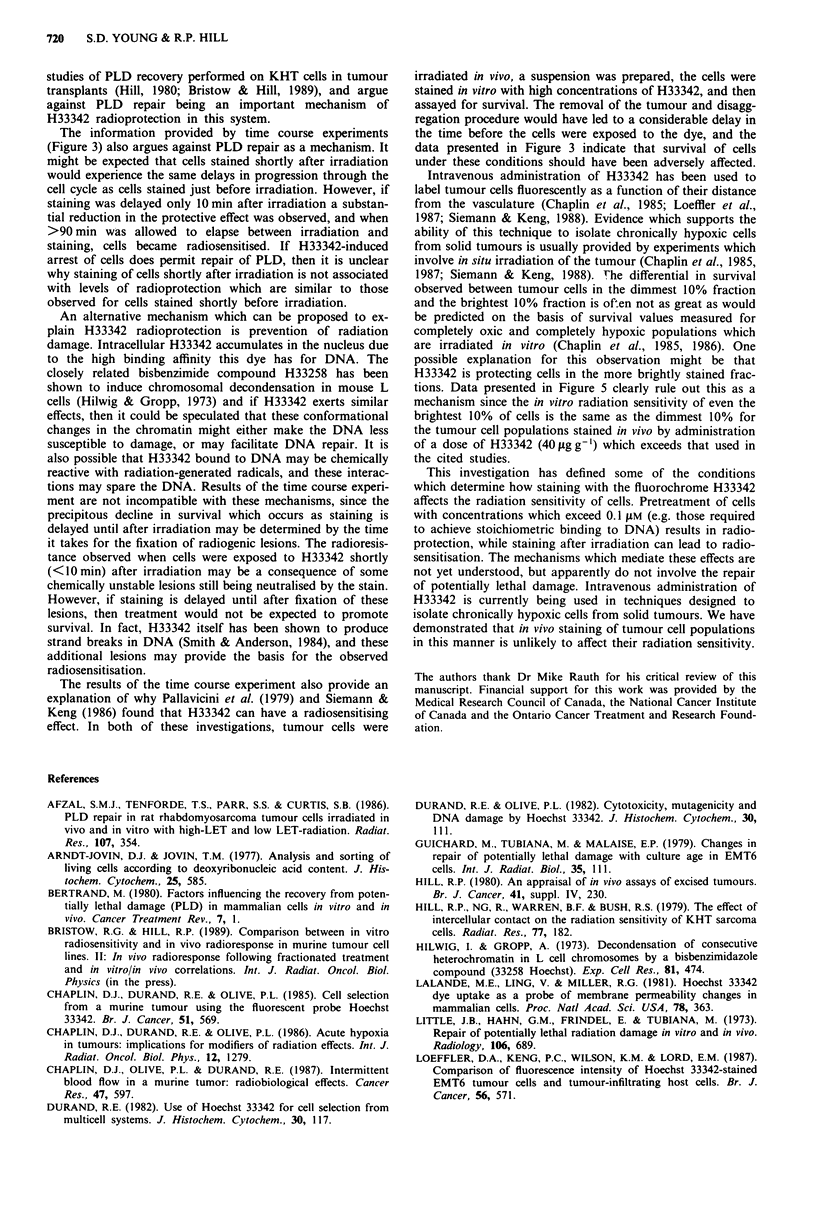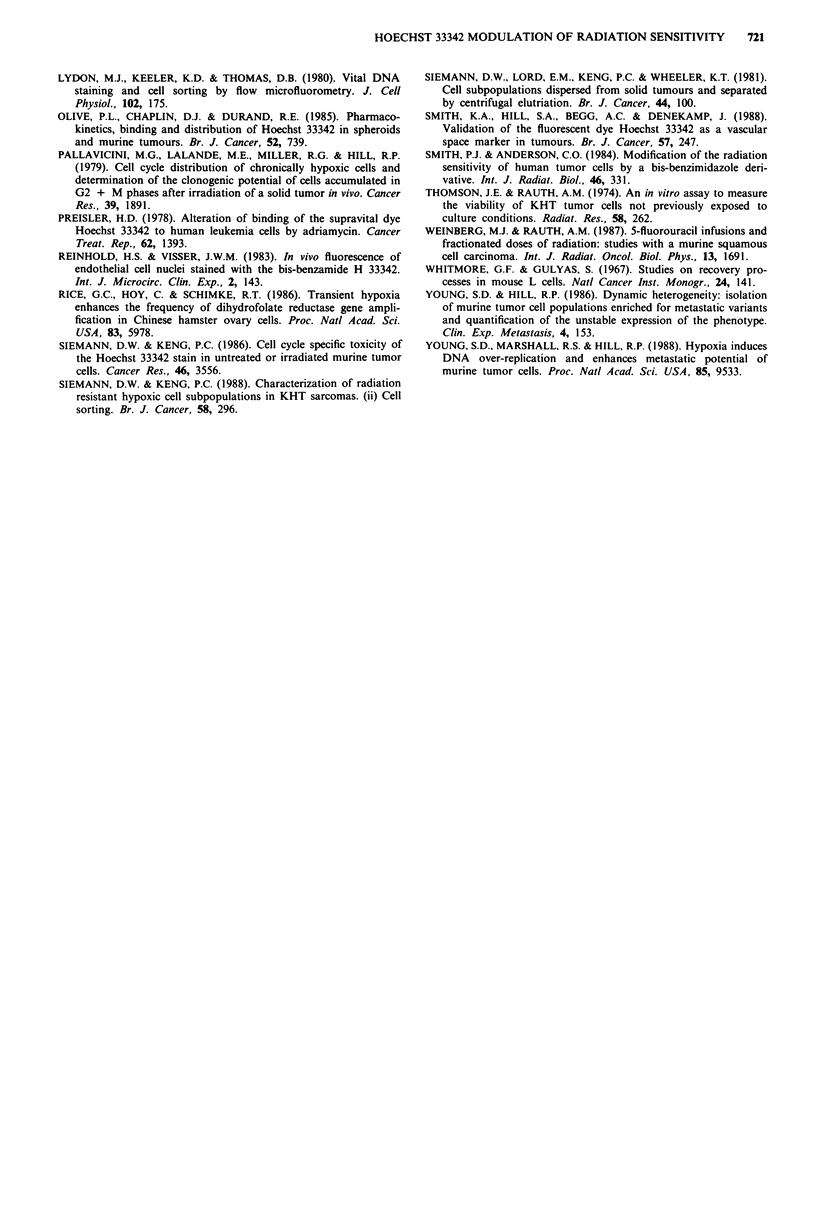# Radiation sensitivity of tumour cells stained in vitro or in vivo with the bisbenzimide fluorochrome Hoechst 33342.

**DOI:** 10.1038/bjc.1989.346

**Published:** 1989-11

**Authors:** S. D. Young, R. P. Hill

**Affiliations:** Physics Division, Ontario Cancer Institute, Toronto, Canada.

## Abstract

The DNA-binding bisbenzimide fluorochrome Hoechst 33342 is being used routinely in radiobiological studies to assess cell kinetic parameters and tumour blood flow. However, there are reports in the literature which indicate that exposure to this compound can affect the radiation sensitivity of tumour cell populations. In this investigation, it was found that staining murine tumour cells in vitro with H33342 at concentrations greater than 0.1 microM before irradiation resulted in radioprotection. The protection factor calculated for fibrosarcoma cells stained with 10 microM H33342 was 1.7. Varying the time between radiation treatment and exposure to the fluorochrome demonstrated that the effect rapidly changed to radiosensitization when staining was performed subsequent to irradiation. Cells in transplanted KHT tumours were stained in vivo by intravenous administration of H33342 to determine whether the radiation sensitivity of these populations might also be modified. Flow cytometric analysis of suspensions prepared from tumours stained in this manner revealed that recovered cells exhibited a greater than 100-fold range in fluorescence intensities. These suspensions were irradiated in vitro and the cells were then fractionated according to fluorochrome content using cell sorting. Little evidence for a radioprotective effect was observed when these subpopulations were assessed for survival, even when tumour-bearing mice were given doses of H33342 which approached the LD50. Further analysis demonstrated that insufficient amounts of the fluorochrome were taken up by cells during in vivo staining to attain levels required for radioprotection. However, our results indicate that the amount of H33342 accumulated by cells may affect the radiation sensitivity of populations exposed to high concentrations of this fluorochrome, such as those required to achieve stoichiometric binding to DNA.


*Br. J. Cancer* (1989), **60**, 715–721 © The Macmillan Press Ltd., 1989

### Radiation sensitivity of tumour cells stained *in vitro* or *in vivo* with the bisbenzimide fluorochrome Hoechst 33342

S.D. Young & R.P. Hill

*Physics Division, Ontario Cancer Institute, 500 Sherbourne Street, Toronto, Ontario, Canada M4X 1K9.*

**Summary** The DNA-binding bisbenzimide fluorochrome Hoechst 33342 is being used routinely in radiobiological studies to assess cell kinetic parameters and tumour blood flow. However, there are reports in the literature which indicate that exposure to this compound can affect the radiation sensitivity of tumour cell populations. In this investigation, it was found that staining murine tumour cells *in vitro* with H33342 at concentrations >0.1 μM before irradiation resulted in radioprotection. The protection factor calculated for fibrosarcoma cells stained with 10 μM H33342 was 1.7. Varying the time between radiation treatment and exposure to the fluorochrome demonstrated that the effect rapidly changed to radiosensitisation when staining was performed subsequent to irradiation. Cells in transplanted KHT tumours were stained *in vivo* by intravenous administration of H33342 to determine whether the radiation sensitivity of these populations might also be modified. Flow cytometric analysis of suspensions prepared from tumours stained in this manner revealed that recovered cells exhibited a >100-fold range in fluorescence intensities. These suspensions were irradiated *in vitro* and the cells were then fractionated according to fluorochrome content using cell sorting. Little evidence for a radioprotective effect was observed when these subpopulations were assessed for survival, even when tumour-bearing mice were given doses of H33342 which approached the $LD_{50}$. Further analysis demonstrated that insufficient amounts of the fluorochrome were taken up by cells during *in vivo* staining to attain levels required for radioprotection. However, our results indicate that the amount of H33342 accumulated by cells may affect the radiation sensitivity of populations exposed to high concentrations of this fluorochrome, such as those required to achieve stoichiometric binding to DNA.

Hoechst 33342 (H33342) is an UV-excitable fluorochrome which has recently found widespread application as a fluorescent probe in studies of tumour biology. At high concentrations (>2 μM) the compound binds stoichiometrically to cellular DNA without inducing significant toxicity (Arndt-Jovin & Jovin, 1977; Durand & Olive, 1982), and hence has been used to sort viable cells according to DNA content (Lydon *et al.*, 1980; Rice *et al.*, 1986; Young *et al.*, 1988). Flow cytometric analysis of cells exposed to this stain has also been used to study the interaction of a chemotherapeutic agent (adriamycin) with leukaemia cells (Preisler, 1978), and to quantitate membrane transport in drug resistant cells (Lalande *et al.*, 1981).

Intravenous injection of H33342 into animals bearing transplanted tumours has been used to examine the morphology of tumour vasculature (Reinhold & Visser, 1983) and the dynamics of tumour blood flow (Chaplin *et al.*, 1987; Smith *et al.*, 1988). In addition, techniques involving diffusion-limited staining of tumour cell populations with H33342 have been developed in an effort to isolate cells from solid tumours as a function of their distance from the vasculature (Chaplin *et al.*, 1985; Olive *et al.*, 1985; Loeffler *et al.*, 1987; Siemann & Keng, 1988) or depth in multi-cellular spheroids (Durand, 1982). These procedures have been used to study tumour cells which reside in hypoxic regions, so that the efficacy of treatments directed at this resistant subpopulation can be specifically evaluated.

The ability of these techniques to isolate tumour cells as a function of their distance from the vasculature is often verified by *in situ* irradiation of the tumour before dissociation (Chaplin *et al.*, 1985, 1987; Siemann & Keng, 1988). Cells in the resulting suspension are fractionated according to dye content using fluorescence-activated cell sorting (FACS) and are then assessed for survival. Under these conditions, cell survival is observed to be inversely related to fluorescence intensity. It has been argued that poorly stained cells are the most radioresistant because they were located at some distance from the vasculature and existed in an hypoxic microenvironment. Cells exhibiting the brightest fluorescence are observed to be relatively radiosensitive because they are located adjacent to capillaries and are well oxygenated. Implicit to this interpretation is the contention that oxygen levels in the microenvironment at the time of irradiation are the major determinants of cell survival, and that the fluorochrome does not affect the radiation sensitivity of the cells. None the less, there are reports in the literature which indicate that H33342 can influence the survival of irradiated cells. Murine fibrosarcoma cells were shown to be radiosensitised by exposure to H33342 (Pallavicini *et al.*, 1979; Siemann & Keng, 1986), while in another study this stain exerted a radioprotective effect on human adenocarcinoma cells (Smith & Anderson, 1984).

The purpose of the current investigation was to establish under what conditions H33342 modifies the survival of irradiated cells, and to determine whether or not the radiation sensitivity of tumour cells stained *in vivo* by intravenous infusion of the fluorochrome is affected.

### Materials and methods

### *Tumour cell lines*

Experiments were conducted using ouabain-resistant KHT-C2-LP1 fibrosarcoma and B16F10-A1 melanoma cells (Young & Hill, 1986), as well as wild-type SCCVII squamous cell carcinoma cells which were obtained from Dr Mike Rauth (Weinberg & Rauth, 1987). Monolayers of tumour cells were grown in plastic flasks containing growth medium which consisted of α-minimal essential medium supplemented with 10% fetal bovine serum and antibiotics. Cultures were propagated by passaging cells twice a week using a trypsinisation procedure. Concentrations of cells in suspensions prepared from monolayers were determined using an electronic particle counter. The plating efficiency (PE) of harvested populations was assessed by transferring known numbers of cells in growth medium on to 100 mm culture plates and counting the number of colonies (containing >50 cells) which arose 10–11 days later. The PE of these cultured cell lines is typically 60–70%.

### *Mice and tumour transplants*

The inbred male C3H/HeJ mice (8–12 weeks old) used in this study were obtained from Jackson Laboratories (Bar

Correspondence: R.P.Hill.
Received 15 March 1989, and in revised form 30 May 1989.

Harbour, Maine, USA) and housed, five per cage, in the specific pathogen-free colony at the Ontario Cancer Institute. Animals were sustained on mouse/rat chow pellets and acidified water *ad libitum*. Tumours were initiated in mice by an intramuscular injection of $2 \times 10^5$ KHT-C2-LP1 cells (in a 0.02 ml volume) into the left hind leg. Tumours were used for experiments when they had attained a size of 1.0–1.2 g, which required ~2 weeks of growth.

### In vitro *staining of tumour cells*

Aliquots of cell suspensions prepared at $\leqslant 10^6$ cells $ml^{-1}$ in growth medium were pelleted and resuspended in an equal volume of growth medium containing Hoechst 33342 (Calbiochem) at the desired concentration (0.01–10 μM). Cells were incubated for 30 min at 37°C in a roller wheel and staining was terminated by washing cells once in chilled growth medium. Samples were kept on ice until used to prevent dye efflux. The PE of stained populations was not adversely affected when cells were exposed to concentrations of H33342 $\leqslant$ 10 μM (data not shown), and these results are consistent with previous reports which have shown that H33342 is relatively non-toxic to mammalian cells (Arndt-Jovin & Jovin, 1977; Durand & Olive, 1982). A non-linear dependency of cellular fluorescence intensity on H33342 concentration has been previously reported (Durand & Olive, 1982), and these observations indicate that staining approaches saturation when concentrations exceed 10 μM. Experiments conducted for this investigation were performed with cells which were stained with $\leqslant$ 10 μM H33342 to avoid significant toxicity.

Plateau-phase monolayers were stained by replacing the culture medium with growth medium containing 10 μM H33342 which had been pre-warmed to 37°C. Flasks were placed on a rocker platform and maintained at 37°C for 30 min, at which time the monolayers were washed twice with phosphate-buffered saline (PBS), pre-warmed growth medium was added, and the cultures returned to a standard incubator. Unstained control populations were treated in an identical manner except they were incubated with dye-free growth medium.

### In vivo *staining of tumour cells*

Mice bearing intramuscular transplants were held in restrainers and infused via the lateral tail vein with either 1 or 10 mg $ml^{-1}$ H33342 freshly dissolved in PBS. The solution was administered through a 27 gauge needle attached to a 1 ml syringe by a length of catheter tubing (PE-20; Clay Adams). After receiving a 100 μl 'loading' volume, the solution was delivered for 30 min at a constant rate (30 μl $min^{-1}$) by an infusion pump. For a 25 g mouse infused with a 1 mg $ml^{-1}$ solution the total dose given was 40 μg $g^{-1}$. Animals were killed 5 min after the infusion and tumours were then removed under aseptic conditions and suspensions were prepared using a combined mechanical and enzymatic (trypsin/DNAse) procedure (Thomson & Rauth, 1974).

### *Purification of cell suspensions by density centrifugation*

Suspensions were enriched for viable tumour cells by centrifugation on a discontinuous Percoll (Pharmacia) density gradient. The gradient was formed in a 60 ml polycarbonate tube and consisted of a 15 ml volume of 70% isotonic Percoll (density 1.086 g $ml^{-1}$) overlayed with an 18 ml volume of 20% Percoll (density 1.028 g $ml^{-1}$). Dilutions of Percoll were made with calcium-, magnesium-free PBS. Cells recovered from tumours were pelleted and resuspended in 4 ml of growth medium and placed on top of the gradient, which was then spun at 450 ***g*** for 20 min at 4°C. Cells which migrated to the interface between the 70% and 20% components were collected by pipette, transferred to a tube containing an excess volume of growth medium, washed and counted. The recovery efficiency of this procedure for dye excluding tumour cells was 70–80%. Suspensions purified in this manner demonstrate enhanced *in vitro* clonogenicity and are much more amenable to flow cytometric analysis since dead cells and debris, which fail to migrate into the gradient, are largely eliminated from the recovered fraction. Red blood cells are also depleted from the sample since their high density causes them to be pelleted on the bottom of the tube.

### *Irradiations*

Tumour cell suspensions (at a concentration of $10^5$ cells $ml^{-1}$) were treated on ice by exposure to $^{60}Co$ γ-rays at a rate of 20 Gy $min^{-1}$. Plateau-phase monolayers (PLDR experiments) were treated by stacking T-25 culture flasks in a large glass beaker and exposing them to the $^{60}Co$ source (Gammacell 220, Atomic Energy Canada Ltd) at room temperature. For all experiments involving radiation treatment, the surviving fraction was calculated by taking into account the PE of an appropriate untreated population.

### *Fractionation of tumour cells according to H33342 fluorescence intensity*

Cell suspensions prepared from tumours resected from mice which had been infused with H33342 were analysed using an EPICS V flow cytometer (Coulter Electronics). Details of the flow cytometric analysis of tumour cells stained with H33342 have been previously published (Young *et al.*, 1988) and involves excitation of cells with 30 mW of 340 nm wavelength laser light and collection of fluorescence emissions at wavelengths >418 nm. A significant proportion of 'contaminating' normal cells in the suspensions could be gated out of the fluorescence analysis because of their small forward-angle light scattering (FALS) signal. Assessment of this excluded subpopulation for *in vitro* clonogenicity after isolation by FACS indicated that only 0.1% of these cells were viable tumour cells. Tumour cell populations were divided into 10 fractions based on fluorescence intensity such that each fraction contained 10% of the tumour cell population. The clonogenicity of cells in the various fractions was determined by sorting known numbers of cells directly on to 60 mm culture plates which contained growth medium and ouabain at a concentration of 0.5 mM. Under these selective growth conditions, only the ouabain-resistant KHT tumour cells will form visible colonies.

### Results

### *Effects of H33342 pretreatment on the survival of irradiated tumour cells*

Preliminary studies examining the influence of H33342 exposure on radiation sensitivity involved staining cultured KHT, B16F10 and SCCVII tumour cells with various concentrations of the dye, immediately treating the suspensions with radiation and plating the cells as soon as possible after treatment. The dose of radiation given to the cells reduced the survival of unstained populations to $\sim 10^{-3}$. The data presented in Figure 1 are from one experiment and demonstrate that for all three cells lines tested, staining with H33342 at concentrations >0.1 μM led to increased levels of survival. For cells stained with the maximum concentration used (10 μM) the surviving fraction was 100 times greater than that for unstained cells.

The radioprotective influence of H33342 was further investigated by producing survival curves for unstained or stained (10 μM) KHT cells (see Figure 2). Comparison of these two curves demonstrates the pronounced radioprotection provided by H33342 staining, which appears to be a dose-modifying effect associated with a protection factor of 1.7.

### *Time course for modulation of radiation sensitivity by H33342 staining*

The available data indicate that H33342 acts as a potent radioprotectant if the cells are exposed to the dye before

**Figure 1** Effect of exposure of murine tumour cells to various concentrations of H33342 on their subsequent radiation sensitivity. Suspensions of cultured tumour cells were incubated in growth medium containing H33342 at the specified concentrations for 30 min at 37°C. The cells were washed once in growth medium, split into two aliquots, and one was irradiated. Both untreated and treated populations were then assayed for *in vitro* clonogenicity, and survival was calculated as the ratio of the plating efficiencies for each dye concentration. □, KHT fibrosarcoma (15 Gy); O, B16F10 melanoma (15 Gy); Δ, SCC VII carcinoma (12.5 Gy).

**Figure 2** Radiation survival curves for unstained (●) and stained (O) KHT cells. Cultured KHT cells were stained with 10 μM H33342 for 30 min, washed once in growth medium, and the suspension divided into aliquots. Each aliquot was treated with a specific dose of radiation and the cells were then assayed for survival. Unstained cells were handled in an identical manner except that they were incubated for 30 min in dye-free medium. Error bars represent ± 1 s.d. of mean survival values obtained from three experiments. $PE_{unstained} = 0.65 \pm 0.02$; $PE_{stained} = 0.56 \pm 0.02$.

irradiation (Figures 1 and 2; Smith & Anderson, 1984). However, two investigations, involving irradiation of KHT tumours *in vivo* followed by disaggregation and *in vitro* staining of the cells, have reported that H33342 adversely affects cell survival (Pallavicini *et al.*, 1979; Siemann & Keng, 1986). Experiments which involved the assessment of the survival of cells which were either stained and irradiated or irradiated and stained were performed to determine whether these conflicting results are due to the sequence of the two treatments. The results of these experiments are presented in Figure 3 and indicate that the timing of H33342 exposure is a critical determinant of cell survival. KHT populations stained up to 5 h before irradiation demonstrated the expected elevated level of survival, but a gradual decline in the radioprotective effect is evident as the time between the termination of staining and irradiation was increased. This may be ributed to a loss of dye from the cells since they were maintained at 37°C during the experiment, and it has been demonstrated that there is dye efflux under these conditions (Durand & Olive, 1982). A precipitous decline in survival was observed when the onset of staining was withheld until after irradiation. Radioprotection was still provided if the delay between treatment and the start of staining was ≤10 min. However, if staining was delayed for longer than about 90 min, survival was lower than that of unstained populations, indicating that H33342 can also produce radiosensitisation. This effect was observed even when staining was delayed for 5 h after irradiation.

### *Effect of delayed plating on the survival of irradiated KHT cells*

It has been established that H33342 delays progression through the cell cycle (Durand & Olive, 1982; Smith & Anderson, 1984), and it is possible that this effect provides additional time for cells to repair radiation damage. Experiments which investigated the capacity of KHT cells to recover from potentially lethal damage (PLD) were therefore conducted to determine whether H33342-induced inhibition of cell cycle progression is a mechanism which can explain the radioprotective properties of the stain. Two types of post-irradiation conditions were used to prevent proliferation. The first involved holding the cells (both unstained and stained) on ice and plating them at various times after irradiation. This procedure has been used to demonstrate PLD repair in cultured cells (Whitmore & Gulyas, 1967), but the survival of KHT cells was not enhanced by delayed plating, even when survival was assayed 5 h post-irradiation (Figure 4).

A second procedure involving the use of plateau-phase monolayers was adopted to evaluate the PLD repair capacity of this KHT subline. KHT cells have been shown to experience plateau-phase growth inhibition (Siemann *et al.*, 1981) and this method is frequently used to study PLD repair in tumour cell lines (Afzal *et al.*, 1986; Guichard *et al.*, 1979; Little *et al.*, 1973). Confluent monolayer cultures (seeded 4 days earlier) were irradiated, maintained at 37°C in nutrient-

**Figure 3** Time course for modification of the radiation sensitivity of KHT cells stained with H33342. Cultured KHT cells were either stained with H33342 (10 μM for 30 min) and then irradiated (negative time values), or irradiated and then stained (positive time values). Cells were maintained in suspension at 37°C during the period between treatments, and were assayed for survival immediately after the treatments were completed. Error bars represent ± 1 s.d. of mean survival values obtained from three experiments.

depleted medium, and the cells were assayed for survival after harvesting at various times for up to 8 h post-irradiation. The cell number recovered from cultures remained constant during the post-irradiation period, and since there was no significant detachment of cells from the monolayers, this observation indicates that proliferation was effectively inhibited. Flow cytometric analysis of the DNA content of cells from these cultures indicated that the majority (~80%) were in $G_1/G_0$-phase (data not shown). None the less, no evidence of a recovery in survival was detected using this approach (see Figure 4).

A third experiment was conducted which also used confluent monolayers, except the culture medium was replaced with fresh growth medium one day before irradiation, and fresh growth medium was also present during the post-irradiation period. The addition of nutrient-rich medium did not appear to release the populations from plateau-phase since there was no detectable increase in the number of cells recovered from the monolayers during the post-irradiation period. This modified procedure has also been used to study repair (Bertrand, 1980), but the results obtained again indicated that the cells did not recover.

In all three experiments, control populations of unirradiated cells (both unstained and stained) were maintained under identical conditions and assayed for *in vitro* clonogenicity. No significant loss of plating efficiency was observed for these populations over the course of these experiments, indicating that the post-irradiation conditions did not adversely affect cell viability. Collectively, these results indicate that this ouabain-resistant KHT subline has little capacity for PLD repair.

### *Survival of KHT cells stained* in vivo *and irradiated* in vitro

The radiation sensitivity of tumour cells stained *in vivo* with H33342 has been assessed in studies involving diffusion-limited staining of tumour transplants to provide evidence that this technique can be used to isolate cells from hypoxic regions of solid tumours (Chaplin *et al.*, 1985; Siemann & Keng, 1988). However, it is generally assumed that the dye does not influence the survival of the irradiated cells. We investigated this issue directly since we had established that pretreatment of mouse tumour cells with H33342 can result in radioprotection.

The *in vitro* radiation sensitivity of KHT cells recovered from solid tumours after *in vivo* staining with H33342 was assessed to determine whether tumour cells with the highest stain uptake were afforded radioprotection. Suspensions were prepared from tumours after the mice had been infused for 30 min with a solution of H33342. These were purified on discontinuous Percoll gradients and the recovered cells were divided into two aliquots, one of which was treated with 15 Gy. These suspensions were then subjected to flow cytometric analysis which revealed that cells stained *in vivo* exhibited a wide range in fluorescence intensities (>100-fold) (see Figure 6 for a representative histogram). Cells were then sorted according to their fluorescence signal into 10 fractions (with each containing 10% of the total population) so that the *in vitro* clonogenicity of cells which had accumulated different amounts of dye could be determined. The PE of cells in the various fractions of the unirradiated aliquot were similar, and ranged from about 30% for the dimmest fraction to about 50% for the most brightly stained cells. The results obtained for cells in the various fractions sorted from irradiated aliquots (see Figure 5) indicate that the survival of cells recovered from mice which had been infused with a 1 mg ml$^{-1}$ solution of H33342 was essentially constant and independent of fluorescence. It can therefore be concluded that even the most brightly stained cells were not radioprotected.

The influence of *in vivo* staining of tumour cells on their subsequent radiation sensitivity was further investigated by infusing tumour-bearing mice with a solution of H33342 which was 10-fold higher in concentration (10 mg ml$^{-1}$). This modification had little effect on the survival of cells in the

**Figure 5** Survival of KHT cells stained *in vivo* and irradiated *in vitro*. KHT cells growing as intramuscular tumours were stained *in vivo* after i.v. infusion of either 1 mg ml$^{-1}$ (●) or 10 mg ml$^{-1}$ (○) H33342. Single cell suspensions were prepared, divided into two aliquots, and one was treated with 15 Gy. Cells in both untreated and treated populations were then sorted into 10 fractions according to their fluorescence intensity and assayed for *in vitro* clonogenicity. The mean surviving fraction measured for three different tumours is provided for both infusion protocols (± 1 s.d.).

**Figure 6** Comparison of fluorescence histograms of KHT cells stained *in vivo* (open) or *in vitro* (filled) with H33342. KHT cells in an intramuscular transplant were stained by infusion of the mouse with a 1 mg ml$^{-1}$ solution of H33342, and the tumour was then disaggregated and the cellular fluorescence examined by flow cytometric analysis. A second suspension was prepared from a KHT tumour resected from a mouse which did not receive any dye, and the recovered cells were stained *in vitro* with specific concentrations of H33342 (0.01 μM or 0.1 μM) for 30 min. These cells, as well as an unstained population, were also subjected to flow cytometric analysis (at the same instrument settings), and the acquired fluorescence histograms superimposed on the histogram obtained for the *in vivo* stained population.

various fractions, although there is a small increase in survival for cells in the brightest fractions relative to that seen for cells stained with a 1 mg ml$^{-1}$ infusion. The amount of H33342 administered with this latter protocol (400 μg g$^{-1}$) exceeds the $LD_{50}$ of the drug for a bolus injection (Olive *et al.*, 1985), and the mice did not tolerate the infusion procedure well. The average modal fluorescence signal of tumour cell populations stained with the 10 mg ml$^{-1}$ protocol was only 5 times greater than the average modal signal recorded for populations stained with 1 mg ml$^{-1}$ (data not shown). This disproportionate increase in fluorescence might be due to a reduced delivery of the compound to the transplants as a result of an H33342-induced reduction in tumour perfusion (Smith *et al.*, 1988).

It may be noted that the relative survival of KHT cells derived from solid tumours after *in vitro* treatment with 15 Gy (Figure 5) is significantly higher than the survival measured for cultured KHT cells treated with the same dose (see Figures 1 and 2). A differential in radiation sensitivity between cells derived from *in vitro* and *in vivo* sources has been previously observed for KHT populations (Hill *et al.*, 1979). This difference was attributed to the effects of 'intercellular contact' which persist for some time after disaggregation of the tumour.

*Relative staining of KHT cells exposed to H33342* in vitro *or* in vivo

An estimate of the relative amount of H33342 which was taken up by tumour cells stained *in vivo* by dye infusion was obtained by comparing the fluorescence histograms of these populations with histograms of tumour cell populations which had been stained *in vitro* with specific concentrations of H33342. Figure 6 provides fluorescence intensity histograms of KHT tumour cells which were stained *in vitro* with either 0.01 μM or 0.1 μM H33342 (30 min exposure), as well as a histogram for unstained tumour cells (signals due to autofluorescence). These are superimposed on a histogram of a KHT tumour cell population which was stained *in vivo* during a 30 min infusion of the tumour-bearing mouse with 1 mg ml$^{-1}$ H33342. A small proportion (~10%) of the *in vivo* stained population exhibit fluorescence values which are similar to the unstained sample. The brightest fraction of cells in the *in vivo* stained population had fluorescence emissions which overlap with those of cells stained *in vitro* with 0.1 μM H33342. Data presented in Figure 1 indicate that radioprotection occurred only when cells were stained ≥0.2 μM H33342 and hence these measurements explain why tumour cells stained *in vivo* with 1 mg ml$^{-1}$ do not demonstrate increased survival after irradiation.

### Discussion

This investigation has established that the fluorochrome H33342 can significantly influence the radiation sensitivity of murine tumour cells. Treatment with non-toxic concentrations of H33342 before irradiation was shown to confer similar levels of radioprotection on cells of three different murine tumour lines. Staining of KHT fibrosarcoma cells with 10 μM H33342 provided a radiation protection factor of 1.7, which is the same as the factor reported for human adenocarcinoma cells stained in a similar manner (Smith & Anderson, 1984).

The mechanism by which H33342 counteracts the damage caused by radiation is not yet understood. Smith & Anderson (1984) proposed that protection of stained populations might be mediated by delaying the progression of cells through the cell cycle, which would permit more time to repair PLD. Flow cytometric studies have demonstrated that exposure to high concentrations of H33342 (>5 μM) results in a temporary accumulation of cells in $G_2$-phase (Durand & Olive, 1982; Smith & Anderson, 1984). H33342 effectively inhibits DNA synthesis, and it is interesting to note that the concentration dependency of H33342 radioprotection (see Figure 1) is very similar to the concentration dependency of H33342-induced inhibition of DNA synthesis (as measured by $^3$H-thymidine incorporation) reported for Chinese hamster V79 fibroblasts (Durand & Olive, 1982). The observed increased survival of stained cells might therefore be a consequence of a prolongation of the time available for cells to repair radiogenic lesions, before they become expressed as lethal damage.

We have examined the PLD repair capacity of KHT tumour cells by subjecting irradiated populations to conditions which inhibited cell proliferation and evaluating survival as a function of time. Studies using this approach indicate that the recovery process nears completion 6–8 h after irradiation (Afzal *et al.*, 1986; Guichard *et al.*, 1979). However, the KHT subline used in these experiments did not show any signs of recovery over an 8 h observation period (see Figure 4). These results are consistent with previous studies of PLD recovery performed on KHT cells in tumour transplants (Hill, 1980; Bristow & Hill, 1989), and argue against PLD repair being an important mechanism of H33342 radioprotection in this system.

The information provided by time course experiments (Figure 3) also argues against PLD repair as a mechanism. It might be expected that cells stained shortly after irradiation would experience the same delays in progression through the cell cycle as cells stained just before irradiation. However, if staining was delayed only 10 min after irradiation a substantial reduction in the protective effect was observed, and when >90 min was allowed to elapse between irradiation and staining, cells became radiosensitised. If H33342-induced arrest of cells does permit repair of PLD, then it is unclear why staining of cells shortly after irradiation is not associated with levels of radioprotection which are similar to those observed for cells stained shortly before irradiation.

An alternative mechanism which can be proposed to explain H33342 radioprotection is prevention of radiation damage. Intracellular H33342 accumulates in the nucleus due to the high binding affinity this dye has for DNA. The closely related bisbenzimide compound H33258 has been shown to induce chromosomal decondensation in mouse L cells (Hilwig & Gropp, 1973) and if H33342 exerts similar effects, then it could be speculated that these conformational changes in the chromatin might either make the DNA less susceptible to damage, or may facilitate DNA repair. It is also possible that H33342 bound to DNA may be chemically reactive with radiation-generated radicals, and these interactions may spare the DNA. Results of the time course experiment are not incompatible with these mechanisms, since the precipitous decline in survival which occurs as staining is delayed until after irradiation may be determined by the time it takes for the fixation of radiogenic lesions. The radioresistance observed when cells were exposed to H33342 shortly (<10 min) after irradiation may be a consequence of some chemically unstable lesions still being neutralised by the stain. However, if staining is delayed until after fixation of these lesions, then treatment would not be expected to promote survival. In fact, H33342 itself has been shown to produce strand breaks in DNA (Smith & Anderson, 1984), and these additional lesions may provide the basis for the observed radiosensitisation.

The results of the time course experiment also provide an explanation of why Pallavicini *et al.* (1979) and Siemann & Keng (1986) found that H33342 can have a radiosensitising effect. In both of these investigations, tumour cells were irradiated *in vivo*, a suspension was prepared, the cells were stained *in vitro* with high concentrations of H33342, and then assayed for survival. The removal of the tumour and disaggregation procedure would have led to a considerable delay in the time before the cells were exposed to the dye, and the data presented in Figure 3 indicate that survival of cells under these conditions should have been adversely affected.

Intravenous administration of H33342 has been used to label tumour cells fluorescently as a function of their distance from the vasculature (Chaplin *et al.*, 1985; Loeffler *et al.*, 1987; Siemann & Keng, 1988). Evidence which supports the ability of this technique to isolate chronically hypoxic cells from solid tumours is usually provided by experiments which involve *in situ* irradiation of the tumour (Chaplin *et al.*, 1985, 1987; Siemann & Keng, 1988). The differential in survival observed between tumour cells in the dimmest 10% fraction and the brightest 10% fraction is often not as great as would be predicted on the basis of survival values measured for completely oxic and completely hypoxic populations which are irradiated *in vitro* (Chaplin *et al.*, 1985, 1986). One possible explanation for this observation might be that H33342 is protecting cells in the more brightly stained fractions. Data presented in Figure 5 clearly rule out this as a mechanism since the *in vitro* radiation sensitivity of even the brightest 10% of cells is the same as the dimmest 10% for the tumour cell populations stained *in vivo* by administration of a dose of H33342 ($40 \mu g g^{-1}$) which exceeds that used in the cited studies.

This investigation has defined some of the conditions which determine how staining with the fluorochrome H33342 affects the radiation sensitivity of cells. Pretreatment of cells with concentrations which exceed 0.1 $\mu$M (e.g. those required to achieve stoichiometric binding to DNA) results in radioprotection, while staining after irradiation can lead to radiosensitisation. The mechanisms which mediate these effects are not yet understood, but apparently do not involve the repair of potentially lethal damage. Intravenous administration of H33342 is currently being used in techniques designed to isolate chronically hypoxic cells from solid tumours. We have demonstrated that *in vivo* staining of tumour cell populations in this manner is unlikely to affect their radiation sensitivity.

The authors thank Dr Mike Rauth for his critical review of this manuscript. Financial support for this work was provided by the Medical Research Council of Canada, the National Cancer Institute of Canada and the Ontario Cancer Treatment and Research Foundation.

### References

AFZAL, S.M.J., TENFORDE, T.S., PARR, S.S. & CURTIS, S.B. (1986). PLD repair in rat rhabdomyosarcoma tumour cells irradiated in vivo and in vitro with high-LET and low LET-radiation. *Radiat. Res.*, **107**, 354.

ARNDT-JOVIN, D.J. & JOVIN, T.M. (1977). Analysis and sorting of living cells according to deoxyribonucleic acid content. *J. Histochem. Cytochem.*, **25**, 585.

BERTRAND, M. (1980). Factors influencing the recovery from potentially lethal damage (PLD) in mammalian cells *in vitro* and *in vivo*. *Cancer Treatment Rev.*, **7**, 1.

BRISTOW, R.G. & HILL, R.P. (1989). Comparison between in vitro radiosensitivity and in vivo radioresponse in murine tumour cell lines. II: *In vivo* radioresponse following fractionated treatment and *in vitro/in vivo* correlations. *Int. J. Radiat. Oncol. Biol. Physics* (in the press).

CHAPLIN, D.J., DURAND, R.E. & OLIVE, P.L. (1985). Cell selection from a murine tumour using the fluorescent probe Hoechst 33342. *Br. J. Cancer*, **51**, 569.

CHAPLIN, D.J., DURAND, R.E. & OLIVE, P.L. (1986). Acute hypoxia in tumours: implications for modifiers of radiation effects. *Int. J. Radiat. Oncol. Biol. Phys.*, **12**, 1279.

CHAPLIN, D.J., OLIVE, P.L. & DURAND, R.E. (1987). Intermittent blood flow in a murine tumor: radiobiological effects. *Cancer Res.*, **47**, 597.

DURAND, R.E. (1982). Use of Hoechst 33342 for cell selection from multicell systems. *J. Histochem. Cytochem.*, **30**, 117.

DURAND, R.E. & OLIVE, P.L. (1982). Cytotoxicity, mutagenicity and DNA damage by Hoechst 33342. *J. Histochem. Cytochem.*, **30**, 111.

GUICHARD, M., TUBIANA, M. & MALAISE, E.P. (1979). Changes in repair of potentially lethal damage with culture age in EMT6 cells. *Int. J. Radiat. Biol.*, **35**, 111.

HILL, R.P. (1980). An appraisal of *in vivo* assays of excised tumours. *Br. J. Cancer*, **41**, suppl. IV, 230.

HILL, R.P., NG, R., WARREN, B.F. & BUSH, R.S. (1979). The effect of intercellular contact on the radiation sensitivity of KHT sarcoma cells. *Radiat. Res.*, **77**, 182.

HILWIG, I. & GROPP, A. (1973). Decondensation of consecutive heterochromatin in L cell chromosomes by a bisbenzimidazole compound (33258 Hoechst). *Exp. Cell Res.*, **81**, 474.

LALANDE, M.E., LING, V. & MILLER, R.G. (1981). Hoechst 33342 dye uptake as a probe of membrane permeability changes in mammalian cells. *Proc. Natl Acad. Sci. USA*, **78**, 363.

LITTLE, J.B., HAHN, G.M., FRINDEL, E. & TUBIANA, M. (1973). Repair of potentially lethal radiation damage *in vitro* and *in vivo*. *Radiology*, **106**, 689.

LOEFFLER, D.A., KENG, P.C., WILSON, K.M. & LORD, E.M. (1987). Comparison of fluorescence intensity of Hoechst 33342-stained EMT6 tumour cells and tumour-infiltrating host cells. *Br. J. Cancer*, **56**, 571.

LYDON, M.J., KEELER, K.D. & THOMAS, D.B. (1980). Vital DNA staining and cell sorting by flow microfluorometry. *J. Cell Physiol.*, **102**, 175.

OLIVE, P.L., CHAPLIN, D.J. & DURAND, R.E. (1985). Pharmacokinetics, binding and distribution of Hoechst 33342 in spheroids and murine tumours. *Br. J. Cancer*, **52**, 739.

PALLAVICINI, M.G., LALANDE, M.E., MILLER, R.G. & HILL, R.P. (1979). Cell cycle distribution of chronically hypoxic cells and determination of the clonogenic potential of cells accumulated in G2 + M phases after irradiation of a solid tumor *in vivo*. *Cancer Res.*, **39**, 1891.

PREISLER, H.D. (1978). Alteration of binding of the supravital dye Hoechst 33342 to human leukemia cells by adriamycin. *Cancer Treat. Rep.*, **62**, 1393.

REINHOLD, H.S. & VISSER, J.W.M. (1983). *In vivo* fluorescence of endothelial cell nuclei stained with the bis-benzamide H 33342. *Int. J. Microcirc. Clin. Exp.*, **2**, 143.

RICE, G.C., HOY, C. & SCHIMKE, R.T. (1986). Transient hypoxia enhances the frequency of dihydrofolate reductase gene amplification in Chinese hamster ovary cells. *Proc. Natl Acad. Sci. USA*, **83**, 5978.

SIEMANN, D.W. & KENG, P.C. (1986). Cell cycle specific toxicity of the Hoechst 33342 stain in untreated or irradiated murine tumor cells. *Cancer Res.*, **46**, 3556.

SIEMANN, D.W. & KENG, P.C. (1988). Characterization of radiation resistant hypoxic cell subpopulations in KHT sarcomas. (ii) Cell sorting. *Br. J. Cancer*, **58**, 296.

SIEMANN, D.W., LORD, E.M., KENG, P.C. & WHEELER, K.T. (1981). Cell subpopulations dispersed from solid tumours and separated by centrifugal elutriation. *Br. J. Cancer*, **44**, 100.

SMITH, K.A., HILL, S.A., BEGG, A.C. & DENEKAMP, J. (1988). Validation of the fluorescent dye Hoechst 33342 as a vascular space marker in tumours. *Br. J. Cancer*, **57**, 247.

SMITH, P.J. & ANDERSON, C.O. (1984). Modification of the radiation sensitivity of human tumor cells by a bis-benzimidazole derivative. *Int. J. Radiat. Biol.*, **46**, 331.

THOMSON, J.E. & RAUTH, A.M. (1974). An *in vitro* assay to measure the viability of KHT tumor cells not previously exposed to culture conditions. *Radiat. Res.*, **58**, 262.

WEINBERG, M.J. & RAUTH, A.M. (1987). 5-fluorouracil infusions and fractionated doses of radiation: studies with a murine squamous cell carcinoma. *Int. J. Radiat. Oncol. Biol. Phys.*, **13**, 1691.

WHITMORE, G.F. & GULYAS, S. (1967). Studies on recovery processes in mouse L cells. *Natl Cancer Inst. Monogr.*, **24**, 141.

YOUNG, S.D. & HILL, R.P. (1986). Dynamic heterogeneity: isolation of murine tumor cell populations enriched for metastatic variants and quantification of the unstable expression of the phenotype. *Clin. Exp. Metastasis*, **4**, 153.

YOUNG, S.D., MARSHALL, R.S. & HILL, R.P. (1988). Hypoxia induces DNA over-replication and enhances metastatic potential of murine tumor cells. *Proc. Natl Acad. Sci. USA*, **85**, 9533.